# Recurrent macerated plaques with severe intertriginous maceration and fissuring: classic clinical presentation of Hailey-Hailey disease

**DOI:** 10.11604/pamj.2026.53.74.50491

**Published:** 2026-02-10

**Authors:** Garima Gupta, Shweta Parwe

**Affiliations:** 1 Department of Panchakarma, Mahatma Gandhi Ayurveda College Hospital and Research Centre, Salod (Hirapur), Datta Meghe Institute of Higher Education and Research, Wardha, Maharashtra, India

**Keywords:** Acantholytic disorders, chronic relapsing dermatosis, clinical image, flexural lesions, groin erosions

## Image in medicine

We report the case of a 70-year-old female patient who presented with chronic, recurrent erosive lesions involving multiple intertriginous regions. The patient had no immediately available medical history at the time of evaluation. Clinical examination revealed symmetric erythematous plaques with maceration, whitish scaling, fissuring, and superficial erosions involving the groin, perineal folds, and proximal thighs. Similar lesions with crusting and post-inflammatory hyperpigmentation were also noted over the lower abdominal fold. The lesions were painful and pruritic, with symptoms exacerbated by friction and perspiration. Based on the characteristic morphology and distribution in flexural areas, a diagnosis of Hailey-Hailey disease (familial benign chronic pemphigus) was made. This genodermatosis is caused by mutations in ATP2C1, leading to defective keratinocyte adhesion and recurrent flares triggered by heat, moisture, and friction. The patient was managed with topical corticosteroids, antifungal agents to control secondary colonization, and barrier-protection measures including moisture reduction and avoidance of friction. Improvement was noted following initiation of therapy.

**Figure 1:**
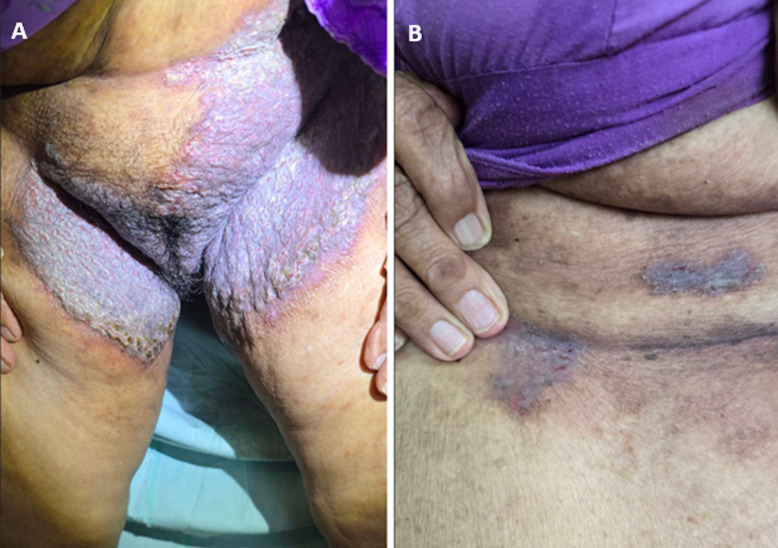
A) extensive macerated, erythematous, and scaly plaques involving the groin and perineum consistent with Hailey-Hailey disease; B) lesions with hyperpigmentation, crusting, and superficial erosions over the lower abdominal fold in the same patient

